# Whole-genome sequence data of the *Listeria monocytogenes* 64_37 isolated from a trout fillet

**DOI:** 10.1016/j.dib.2025.112301

**Published:** 2025-11-20

**Authors:** Aleksandra Slavko, Violeta Pemaj, Eleftherios H. Drosinos, Panagiotis N. Skandamis, Kathrin Rychli, Konstantinos Papadimitriou

**Affiliations:** a Department of Food Science and Technology, University of the Peloponnese, Antikalamos 24100, Greece; b Laboratory of Food Quality Control and Hygiene, Department of Food Science and Human Nutrition, Agricultural University of Athens, Iera Odos 75, Athens 118 55, Greece; c Centre for Food Science and Veterinary Public Health, Clinical Department for Farm Animals and Food System Science, University of Veterinary Medicine, Veterinaerplatz 1, 1210 Vienna, Austria

**Keywords:** Listeria monocytogenes, Trout fillet, Whole-genome sequencing, Virulence, Antimicrobial resistance

## Abstract

*Listeria monocytogenes* is a major foodborne pathogen causing listeriosis disease in humans. Fish is a product that can serve as a transmission source. Here we present the whole-genome sequence (WGS) of *L. monocytogenes* strain 64_37 isolated from a trout fillet. Initial de novo assembly of Illumina raw reads produced 40 contigs of 3,016,736 bp and a GC % content of 37.85 %. Further reference-based assembly resulted in a high-quality genome consisting of one scaffold with a total length 2,957,126 bp and a completeness of 99.99 %. A *rep26*-type replicon plasmid was detected. MLST analysis assigned strain 64_37 to serogroup IIa and sequence type (ST) 204 of clonal complex (CC) 204. Functional annotation using the Rapid Annotation using Subsystem Technology (RAST) server revealed 2,949 coding sequences and 41 RNA sequences. Pan-genome analysis identified 88 unique genes in strain 64_37. Investigation of the pathogenic potential of *L. monocytogenes* 64_37 revealed the presence of multiple virulence genes. Investigation of the presence of antimicrobial resistance (AMR) genes suggested resistance to lincosamide, cationic peptides, fosfomycin and fluoroquinolone. This data provides an overview of pathogenic potential of *L. monocytogenes* 64_37 and can be used to further shed light on its presence in fish products.

Specifications Table

**Table:** 

Subject	Biology
Specific subject area	Genome sequencing and in silico analysis.
Type of data	Genome sequence, tables and figures.
Data collection	*Listeria monocytogenes* 64_37 was isolated in 1997 in Austria from trout fillet, the genomic DNA was obtained from the pure culture and sequenced by Novogene (Novogene, Cambridge, UK) on Illumina NovaSeq 6000 platform (Illumina, San Diego, CA, USA). Standard procedures were followed, including library preparation, and 2 × 250 bp paired-end sequencing. The raw reads were checked for their quality with FastQC v0.11.9, trimmed using Cutadapt v4.5 and assembled de novo using the Unicycler v0.5.1. The quality of the de novo assembly of *L. monocytogenes* was checked with Quast v5.0.2 and the final reference-based assembly was generated using Ragout v2.3. Genome completeness and contamination were assessed with CheckM2. PlasmidFinder v 2.1 was used for plasmid detection, and the genome was aligned against the reference sequence with MAUVE. DNAPlotter was employed to visualize the pseudochromosome map of the *L. monocytogenes* 64_37. The multilocus sequence typing (MLST) was performed with the MLST locus scheme for *L. monocytogenes* using BIGSdb-Pasteur. Functional annotation was conducted using the RAST server. Pan-genome analysis was performed using Prokka v1.14.5 and Roary v3.13.0. The presence of virulence and antimicrobial resistance (AMR) genes was determined with ABRicate v1.0.1 against the Virulence Factor Database (VFDB), ResFinder and the Comprehensive Antibiotic Resistance Database (CARD).
Data source location	Laboratory of Food Quality Control and Hygiene, Department of Food Science and Human Nutrition, Agricultural University of Athens, 118 55 Athens, Greece.
Data accessibility	Raw sequence data of *L. monocytogenes* 64_37 was deposited in the Sequence Read Archive (SRA) under accession number SRR35782905 (https://www.ncbi.nlm.nih.gov/sra/?term=SRR35782905): WGS of *Listeria monocytogenes* 64_37 isolated from trout fillet.

## 1 Value of the Data

• *Listeria monocytogenes* is an important foodborne pathogen commonly associated with fish and fish products. The whole genome sequence of the strain isolated from trout fillet will provide valuable insights into its adaptation in this environment.
• The data presented in this manuscript is useful for researchers working in the fields of microbial ecology and food safety.
• The genomic information of *L. monocytogenes* 64_37 presented in this study can facilitate comparative and evolutionary analyses among strains isolated from different food sources.

## 2 Background

Fish and fish products are important components of the human diet. Despite being excellent sources of nutrients, fish can also serve as a source of pathogenic bacteria, including *Listeria monocytogenes* [1]. Strains of *L. monocytogenes* have previously been isolated from salmon and trout, among them several have been linked to large multicountry outbreaks of listeriosis, a severe disease with high fatality rate [2,3]. Therefore, whole-genome sequencing (WGS) of *L. monocytogenes* strains isolated from fish and fish products is important for understanding of their genetic characteristics, virulence potential and antimicrobial resistance.

## 3 Data Description

**Fig. 1:**
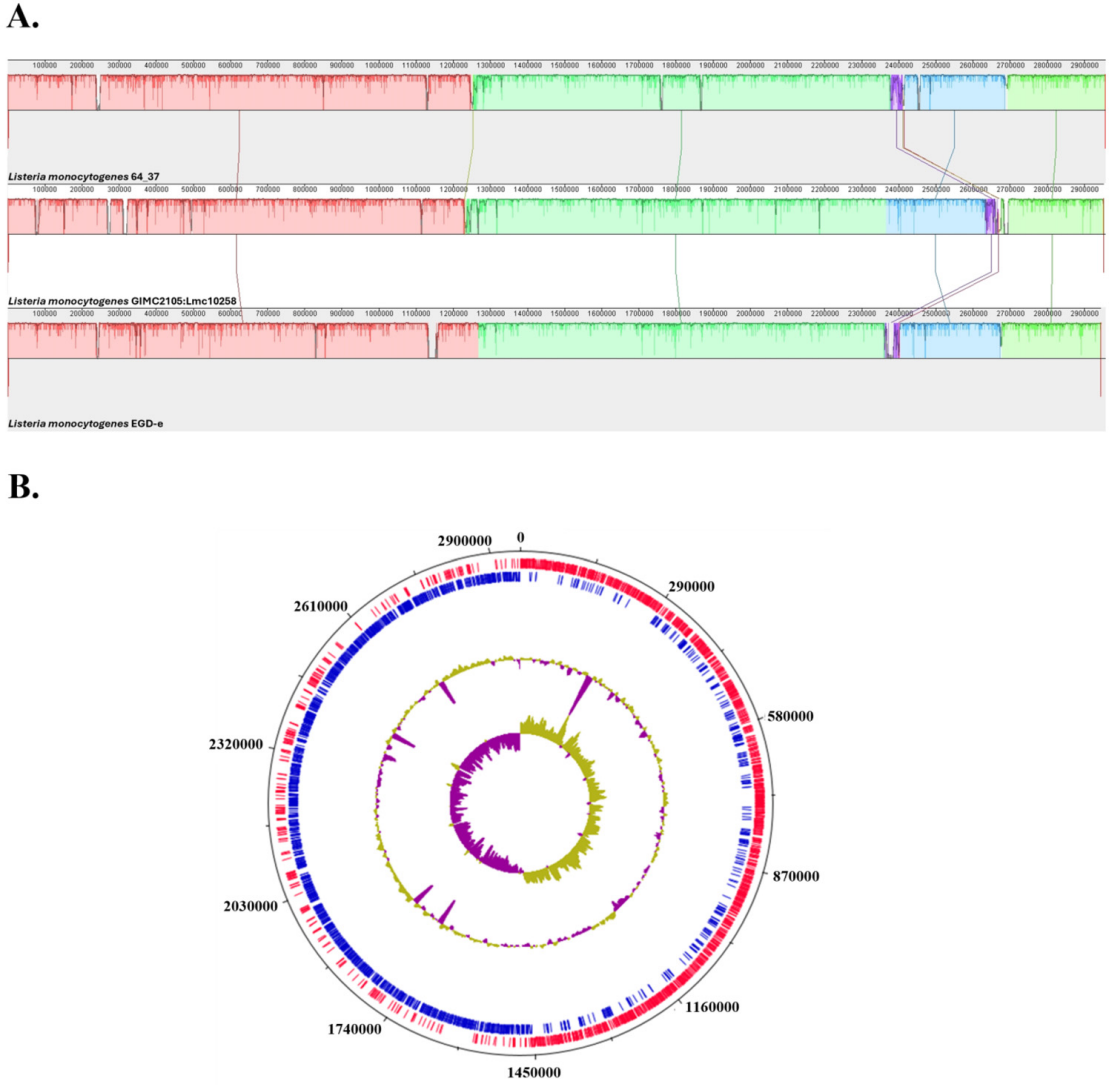
(A) Alignment of *L. monocytogenes* strain 64_37 against the reference chromosome of *L. monocytogenes* EGD-e and fish derived isolate GIMC2105:Lmc10258 generated using MAUVE. The horizontal axis indicates genomic position (bp). Syntenic regions are represented as local colinear blocks (LCBs) in different colors, with block height corresponding to the level of local nucleotide identity between the genomes. White gaps between LCBs represent regions of genomic divergence between the strains. (B) Circular genome map of the *L. monocytogenes* 64_37 generated with DNAPlotter.

Here, we present a high-quality genome of *L. monocytogenes* strain 64_37 isolated from a trout fillet. The initial de novo assembly of the paired-end Illumina sequencing reads resulted in 40 contigs with total length of 3,016,736 bp and a GC % content of 37.85 %. The final reference-based assembly comprised one scaffold of total length 2,957,126 bp with completeness of 99.99 % and contamination level of 1.07 %. In addition, a *rep26*-type replicon plasmid was detected. MLST analysis assigned strain 64_37 to serogroup IIa and ST204 of CC204. Alignment of strain 64_37 with the reference strain *L. monocytogenes* EGD-e and a fish-derived complete genome of *L. monocytogenes*, together with the circular representation of its genome, are presented in Fig. 1.

Genome annotation performed with Rapid Annotation using Subsystem Technology (RAST) server identified 2,949 coding sequences and 41 RNA sequences (Table 1).

**Table 1:** Characteristics of the genome assembly of *L. monocytogenes* 64_37 according to the RAST server.

Sequence trait	Value
Size (bp)	2,957,126
GC Content (%)	37.8
Number of Contigs	1
Number of Subsystems	266
Number of Coding Sequences	2,949
Number of RNAs	41

**Fig. 2:**
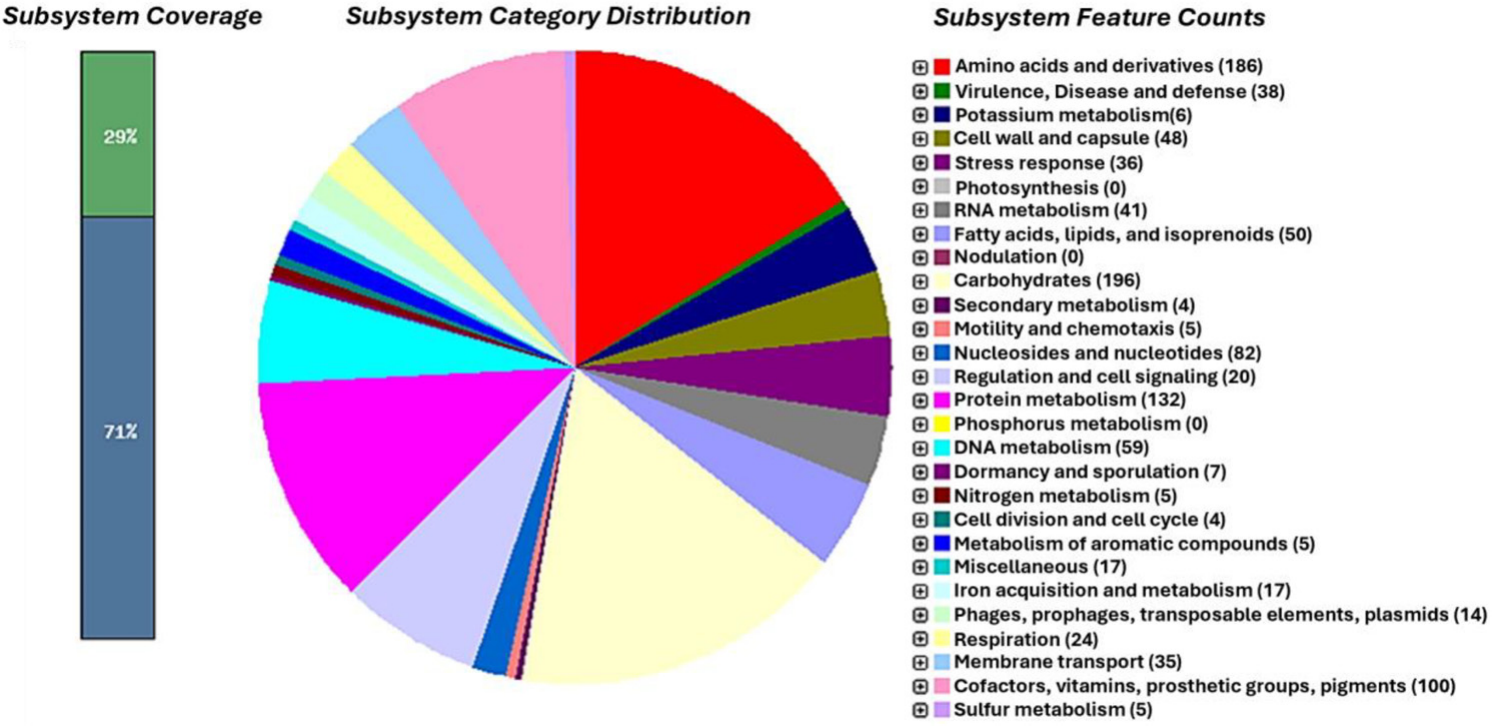
Protein encoding genes of the *L. monocytogenes* 64_37 assigned to subsystems categories according to the RAST server.

Among the annotated features, the most represented categories were related to carbohydrates (196), amino acids and derivatives (186), protein metabolism (132), cofactors, vitamins, prosthetic groups and pigments (100) as well as nucleosides and nucleotides (82) (Fig. 2).

**Fig. 3:**
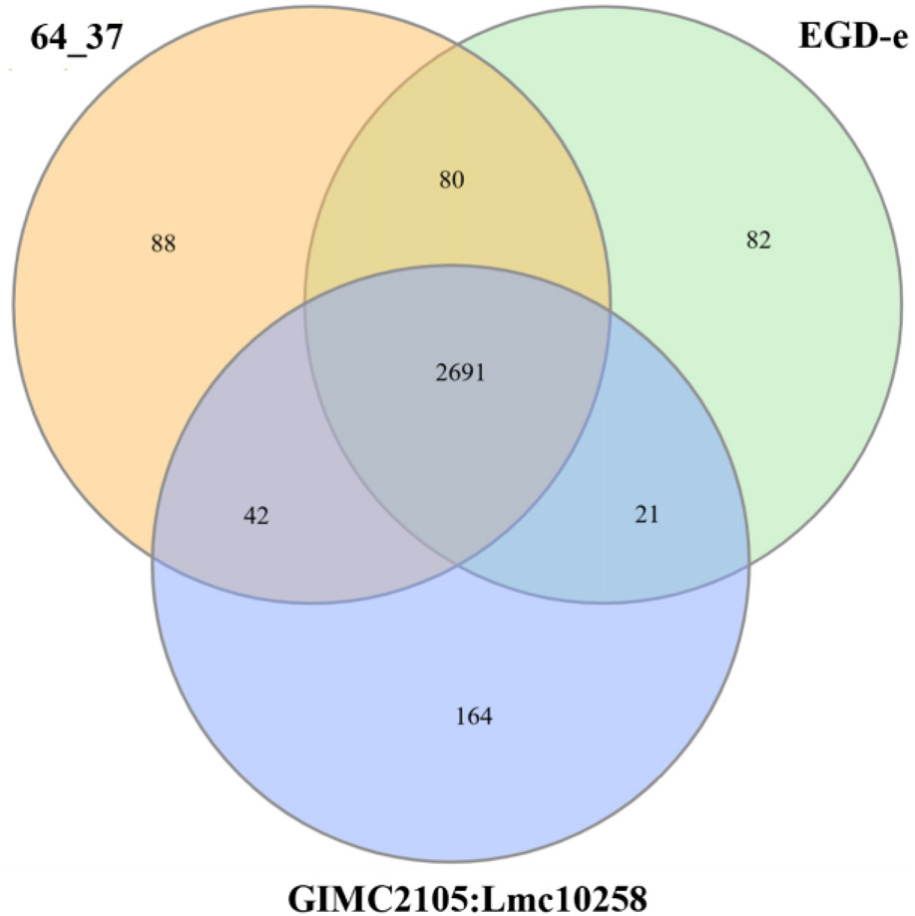
Venn diagram showing the distribution of shared and unique genes among *L. monocytogenes* strains.

Pan-genome comparison between *L. monocytogenes* 64_37, EGD-e and the fish-derived *L. monocytogenes* isolate GIMC2105:Lmc10258 revealed that all genomes shared 2,691 core genes, whereas strain 64_37 possessed 88 unique genes, primarily encoding hypothetical proteins (Fig. 3).

The investigation of the pathogenic potential of *L. monocytogenes* 64_37 showed the presence of multiple virulence genes (Table 2).

**Table 2:** Virulence genes identified in *L. monocytogenes* 64_37.

Gene	Product
*prfA* *plcA* *hly* *mpl* *actA* *plcB* *clpC* *pdgA* *inlA* *inlB* *inlC* *inlF* *inlK* *inlJ* *lntA* *iap/cwhA* *hpt* *lplA1* *clpE* *aut* *oatA* *lap* *lapB* *fbpA* *lspA* *lpeA* *bsh* *prsA2* *clpP* *gtcA* *ami*	Listeriolysin positive regulatory protein Phosphatidylinositol-specific phospholipase c Listeriolysin O precursor Zinc metalloproteinase precursor Actin-assembly inducing protein precursor Phospholipase C Endopeptidase Clp ATP-binding chain C Peptidoglycan N-deacetylase Internalin A Internalin B Internalin C Internalin F Internalin K Internalin J *Listeria* nuclear targeted protein A P60 extracellular protein invasion associated protein Iap Hexose phosphate transport protein Lipoate protein ligase ATP-dependent protease Autolysin Peptidoglycan O-acetyltransferase *Listeria* adhesion protein Lap *Listeria* adhesion protein LapB Fibronectin-binding protein Signal peptidase II Lipoprotein promoting cell invasion Bile salt hydrolase Post translocation chaperone PrsA2 ATP-dependent Clp protease proteolytic subunit Wall teichoic acid glycosylation protein GtcA Autolysin amidase adhesin

Screening for antimicrobial resistance genes revealed the presence of *lin, mprF, fosX* and *norB* suggesting resistance of the strain to lincosamide, cationic peptides, fosfomycin and fluoroquinolone, respectively [4].

## 4 Experimental Design, Materials and Methods

*Listeria monocytogenes* 64_37 was isolated in 1997 in Austria from a trout fillet. The culture was grown in BHI broth (Oxoid, Basingstoke, United Kingdom) at 37 °C. High quality genomic DNA was extracted with the NucleoSpin DNA RapidLyse kit (MACHEREY-NAGEL, Düren, Germany) according to the manufacturer’s instructions.

WGS of the *L. monocytogenes* 64_37 was performed by Novogene (Novogene, Cambridge, UK) using the Illumina NovaSeq 6000 platform (Illumina, San Diego, CA, USA). Standard procedures were followed, including library preparation, 2 × 250 bp paired-end sequencing and subsequent bioinformatics analysis. Raw sequencing reads were assessed for quality with FastQC v0.11.9 [5], trimmed using Cutadapt v4.5 [6] and assembled de novo using the Unicycler v0.5.1 [7]. The quality of the de novo assembly of *L. monocytogenes* was assessed with Quast v5.0.2 [8]. The final reference-based assembly was generated with Ragout v2.3, with the *L. monocytogenes* EGD-e reference genome (NC_003210.1) [9]. The *L. monocytogenes* EGD-e strain was chosen as the reference genome as it represents the official reference of ReSeq and GenBank for the species. Genome completeness and contamination were evaluated with CheckM2 [10]. PlasmidFinder v2.1 was used to screen for plasmids [11]. The genome assembly was aligned against the EGD-e reference sequence and one randomly picked fish-derived strain GIMC2105:Lmc10258 (NZ_CP127191.1) using MAUVE [12]. The circular genome map was generated with DNAPlotter [13]. The MLST profile of *L. monocytogenes* strain 64_37 was determined using the MLST locus scheme for *L. monocytogenes* at BIGSdb-Pasteur [14]. Functional annotation was performed using the RAST server [15]. The pan-genome of three *L. monocytogenes* strains 64_37, EGD-e, and GIMC2105:Lmc10258 was analyzed using Prokka v1.14.5 and Roary v3.13.0 [16]. The Venn diagram was constructed using the InteractiVenn [17]. The presence of virulence and AMR genes in genome was determined with ABRicate v1.0.1 against the Virulence Factor Database (VFDB), ResFinder and the Comprehensive Antibiotic Resistance Database (CARD) [[18], [19], [20], [21]]. All bioinformatics tools used in this study were run with default parameters.

## Limitations

None.

## Ethics Statement

The authors have read and followed the ethical requirements for publication in Data in Brief and confirm that the current work does not involve human subjects, animal experiments, or any data collected from social media platforms.

## CRediT Author Statement

**Aleksandra Slavko:** Formal analysis, Writing – original draft; **Violeta Pemaj:** Formal analysis, Writing – original draft; **Eleftherios H. Drosinos:** Writing – original draft; **Panagiotis N. Skandamis:** Writing – original draft; **Kathrin Rychli:** Conceptualization, Writing – original draft; **Konstantinos Papadimitriou:** Supervision, Conceptualization, Methodology, Data curation, Writing – original draft.

## Data Availability

SRAWGS of Listeria monocytogenes 64_37 isolated from trout fillet (Original data).
